# Structural and optical characterizations of InPBi thin films grown by molecular beam epitaxy

**DOI:** 10.1186/1556-276X-9-24

**Published:** 2014-01-13

**Authors:** Yi Gu, Kai Wang, Haifei Zhou, Yaoyao Li, Chunfang Cao, Liyao Zhang, Yonggang Zhang, Qian Gong, Shumin Wang

**Affiliations:** 1 State Key Laboratory of Functional Materials for Informatics, Shanghai Institute of Microsystem and Information Technology, Chinese Academy of Sciences, Shanghai 200050, China; 2 Department of Microtechnology and Nanoscience, Chalmers University of Technology, Gothenburg 41296, Sweden

**Keywords:** InPBi, HRXRD, Absorption, Photoluminescence, Molecular beam epitaxy

## Abstract

InPBi thin films have been grown on InP by gas source molecular beam epitaxy. A maximum Bi composition of 2.4% is determined by Rutherford backscattering spectrometry. X-ray diffraction measurements show good structural quality for Bi composition up to 1.4% and a partially relaxed structure for higher Bi contents. The bandgap was measured by optical absorption, and the bandgap reduction caused by the Bi incorporation was estimated to be about 56 meV/Bi%. Strong and broad photoluminescence signals were observed at room temperature for samples with *x*_Bi_ < 2.4%. The PL peak position varies from 1.4 to 1.9 μm, far below the measured InPBi bandgap.

## Background

Group III-V semiconductors containing small amounts of bismuth (Bi), popularly known as ‘dilute bismide,’ attracted great attention in the past decade. Bismuth is the largest and the heaviest group V element with its isoelectronic energy level that resides in the valence band of most III-V materials. Incorporation of a small amount of Bi atoms in a common III-V compound is expected to lead to a large bandgap reduction [1] and strong spin-orbit splitting [2]. This provides a new degree of freedom to engineering the band structure for potential optoelectronic and electronic device applications. Under such conditions, it is expected that troublesome hot-hole-induced Auger recombination and inter-valence band absorption (IVBA) processes can be suppressed leading to high efficiency and temperature insensitive lasers for optical communications [3]. Most published literatures so far focus on growth and material properties of GaAsBi with improving quality, making GaAsBi closer to device applications. GaAsBi light-emitting diodes (LEDs) [4] and optically pumped [5] and electrically injected [6] laser diodes have been demonstrated recently.

Group III-V semiconductor phosphides are important materials for optoelectronic devices working at visible and near-infrared wavelength range [7,8]. The incorporation of Bi into InP can further extend transition wavelengths for optoelectronic devices with aforementioned improved device performances as a result of the suppressed Auger recombination and IVBA processes. Berding et al. theoretically compared InPBi, InAsBi, InSbBi, and HgCdTe, and pointed out that InPBi was much more robust than the others, thus making it as a promising candidate for infrared applications. However, their calculations also showed that InPBi was very difficult to synthesize due to a larger miscibility gap than that of InAsBi and InSbBi [9]. So far, a few works on the optical studies of InP/Bi where the incorporated Bi is only in the doping level [10,11] were reported. The spectroscopy reveals rich sharp transitions at energy levels close to the InP bandgap at low temperatures.

In this work, we investigate the structural and optical properties of InPBi with Bi composition in the range of 0.6% to 2.4%. The Bi-induced bandgap reduction of around 56 meV/Bi% is obtained. Strong and broad photoluminescence (PL) signals have been observed at transition energy much smaller than the InPBi bandgap.

## Methods

The samples were grown on (100) semi-insulating InP substrates by V90 gas source molecular beam epitaxy (GSMBE). Elemental In and Bi and P_2_ cracked from phosphine were applied. After the surface oxide desorption of InP substrate at 524°C, a 75-nm undoped InP buffer was grown at 474°C, the normal growth temperature of InP. Then the growth temperature was decreased significantly for InPBi growth. Both the Bi/P ratio and the growth temperature were adjusted to achieve InPBi with various Bi compositions. The thickness of the InPBi epi-layers was kept around 430 nm. An InP reference sample was also grown at the low temperature.

After the growth, the Bi compositions were determined by Rutherford backscattering spectrometry (RBS) with 2.275 MeV ^4^He^2+^ ions. The structural qualities were characterized by a Philips X’pert MRD high-resolution x-ray diffractometer (HRXRD) equipped with a four-crystal Ge (220) monochromator (Philips, Amsterdam, Netherlands). The PL and absorption spectra were measured using a Nicolet Magna 860 Fourier transform infrared (FTIR) spectrometer (Thermo Fisher Scientific Inc., Waltham, MA, USA), in which a liquid-nitrogen cooled InSb detector and a CaF_2_ beam splitter were used. A diode-pumped solid-state (DPSS) laser (*λ* = 532 nm) was used as the excitation source for PL measurements, and the double modulation mode was used to eliminate the mid-infrared background radiation beyond 2 μm [12]. For the low-temperature PL measurements, the samples were mounted into a continuous-flow helium cryostat, and the temperature was controlled from 8 to 300 K by a Lake Shore 330 temperature controller (Lake Shore Cryotronics, Inc., Westerville, OH, USA).

## Results and discussions

**Figure 1:**
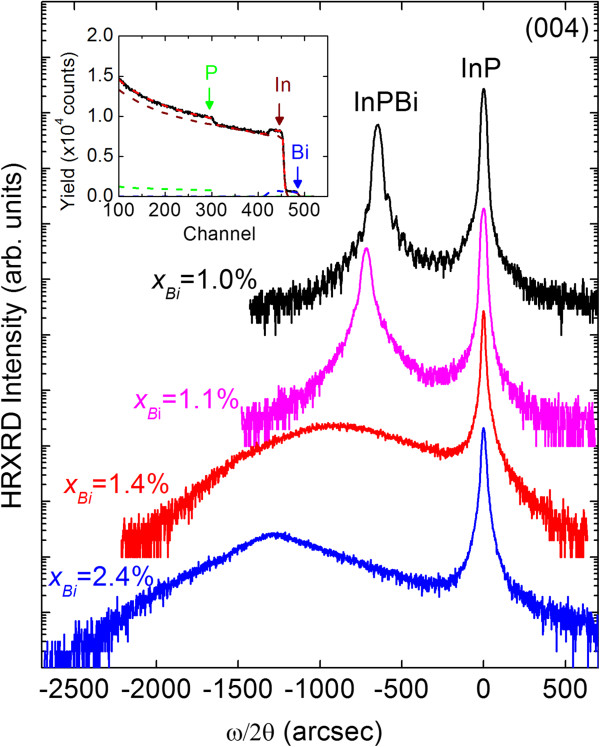
**HRXRD (004) scan curves of InPBi samples with various Bi compositions.** The inset shows the RBS spectrum from the InPBi film with *x*_Bi_ = 1.4% (solid line). The simulated spectrum and the contributions of Bi, In, P are also contained (dashed lines).

The Bi incorporation was examined by RBS measurements as shown in the inset of Figure 1, and the Bi concentrations were deduced from the simulations. For all the InPBi samples with various Bi compositions, two main peaks are observed in the HRXRD *ω*/2*θ* scan curves in the (004) reflection direction as shown in Figure 1. The narrower peak with a stronger intensity corresponds to the InP buffer layer and substrate for each sample, while the peak on the left side corresponds to InPBi epi-layer. Asymmetric (224) reflections were performed to obtain the exact lattice mismatch between the epi-layer and the substrate. Then the strain relaxation and lattice constant of each sample were obtained, assuming the same Poisson ratio for InPBi and InP. The relaxation degree increased to about 35% for the sample with the highest Bi content, while the sample with the least Bi composition is nearly fully strained. As the Bi content increases, the HRXRD peak intensity of InPBi is reduced and the peak width increases from about 46 to 580 arcsec due to the partial lattice relaxation. Using the Vegard’s law and the lattice constant value of InP 5.8688 Å, the average lattice constant of InBi binary alloy is calculated to be 7.292 Å, which is much larger than the former reports of 6.639 Å [13], 6.686 Å [14], or 7.024 Å [15].

**Figure 2:**
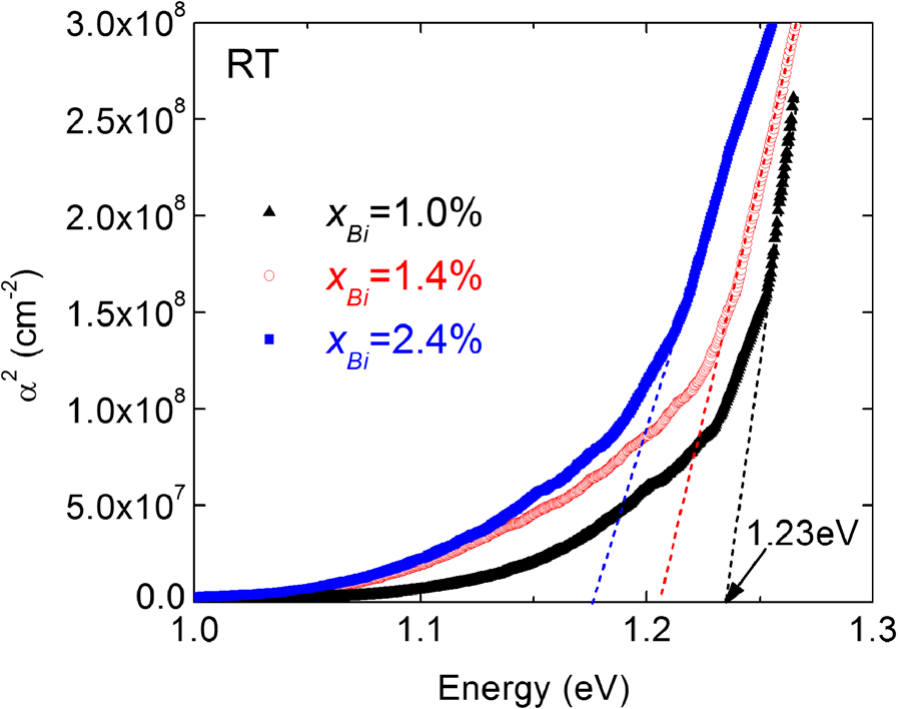
**Square of absorption coefficient of InPBi samples.** Square of absorption coefficient of InPBi samples with various Bi compositions as a function of photon energy at room temperature.

**Figure 3:**
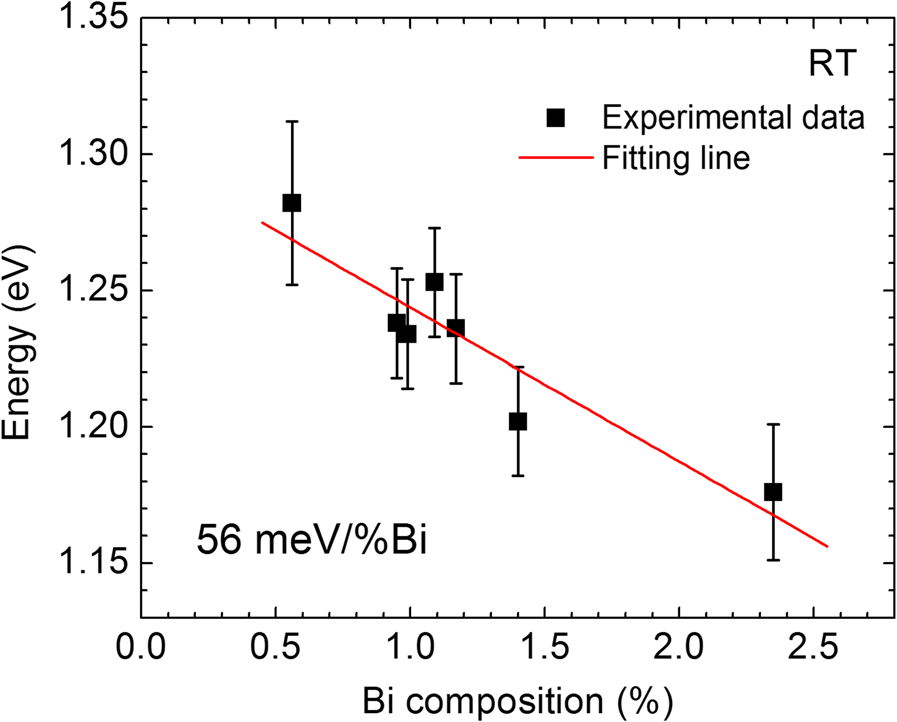
**Bandgap energy of InPBi measured from absorption spectra as a function of Bi composition.** The error bars of the experimental data are labeled. The solid line is the fitting line of the experimental data.

Figure 2 shows square of absorption coefficient of InPBi films with various Bi compositions as a function of photon energy at room temperature (RT). The band edge of the InPBi film shifts to longer wavelengths as the Bi composition increases, revealing a reduction of the bandgap energy. The bandgap value is obtained from the linear extrapolation of the rising part for each sample [16] and shown in Figure 3, where the error bars are also labeled. By using the linear fitting of the experimental data, the Bi-induced bandgap reduction of about 56 meV/%Bi is obtained, which is smaller than the value of 88 meV/%Bi for GaAsBi [1] close to 55 meV/%Bi for InAsBi [15], but larger than 23 meV/%Bi for InSbBi [17].

**Figure 4:**
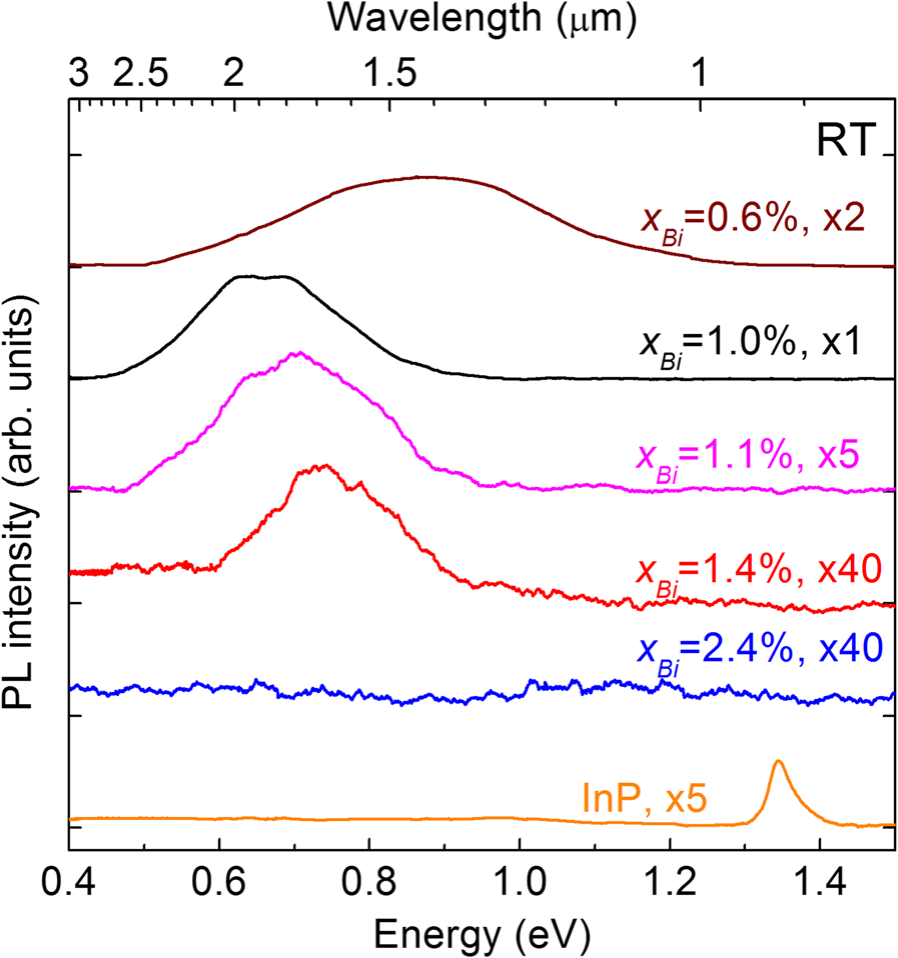
**PL spectra of InPBi films with various Bi compositions at RT.** The PL spectrum of InP reference sample is also shown.

Figure 4 shows the PL spectra of InPBi films with Bi composition *x*_Bi_ from 0.6% to 2.4% at RT. Strong and broad PL peaks are observed for the samples, except for the sample with the highest Bi composition. The PL peak energy first shifts from 0.9 eV (1.4 μm) to 0.65 eV (1.9 μm), when *x*_Bi_ increases from 0.6% to 1.0%, and then turns back for the samples with a higher *x*_Bi_, but in all cases far from the bandgap energy. On the other hand, the InP reference sample only shows one PL peak at around 1.34 eV (0.93 μm) corresponding to the band-to-band transition. The InPBi sample with *x*_Bi_ = 0.6% shows a very broad PL envelope from about 1.2 eV (1 μm) to 0.5 eV (2.5 μm), with a peak wavelength at around 0.9 eV (1.4 μm). The sample with *x*_Bi_ = 1.0% reveals the longest PL wavelength (peak at about 1.9 μm) and the strongest intensity. As the Bi composition further increases, the PL wavelength starts to blueshift and the PL intensity decreases. For the sample with 1.4% Bi, the PL peak is blueshifted to around 0.73 eV (1.7 μm) and the PL intensity is weakened to about 1/40 of the sample with the strongest PL intensity. No PL signal was detected for the sample with 2.4% Bi. The clear RT PL signals far from the InPBi bandgap are unexpected. The Bi incorporation into GaAs was found to induce shallow localized states associated with Bi clusters above the top of the GaAs valence band due to the valence band anticrossing interaction, thus causing the red shift of PL [1,18]. In addition, the Bi in InP with a doping level was found to act as isoelectronic impurities and revealed rich spectroscopic information near the bandgap of InP (1.3 to 1.4 eV) at low temperatures [10,11]. However, the effects of cluster localization and isoelectronic impurities both introduce the PL peak red shift near the InP bandgap energy, in contrast to the PL signals observed from the middle of the bandgap.

**Figure 5:**
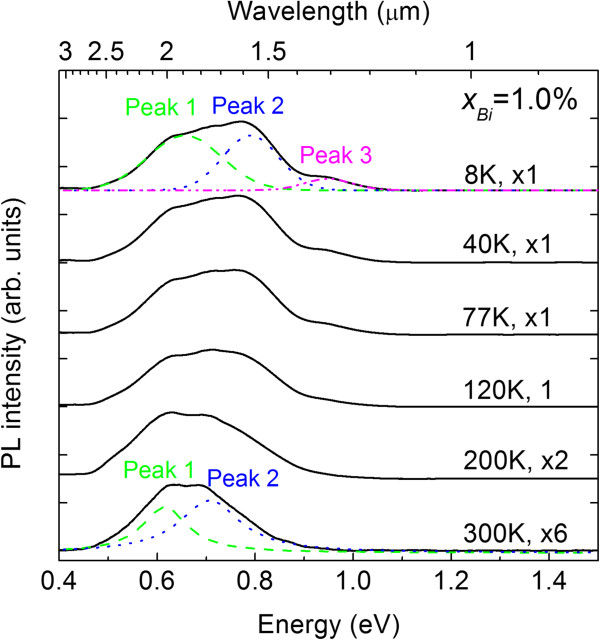
**PL spectra of the InPBi sample with 1.0% Bi at various temperatures.** The overlapped multi-peaks obtained by using Gaussian fitting are shown as the dashed and dotted lines for the cases of 8 and 300 K, and the multi-peaks of PL spectra at other temperatures were also obtained similarly.

**Figure 6:**
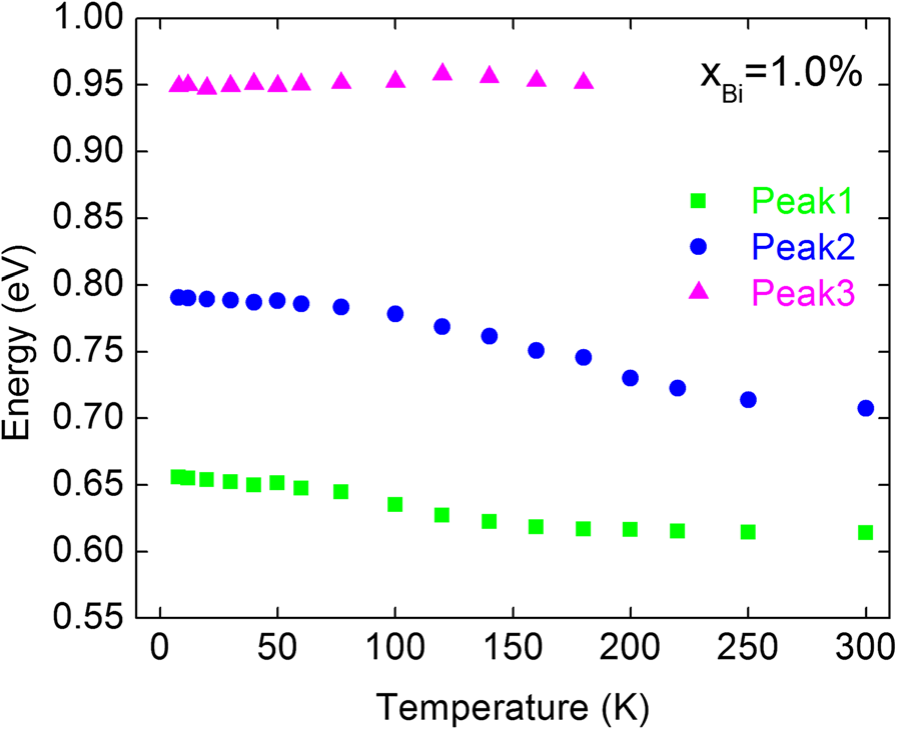
**PL energies of the multi-peaks at various temperatures for the InPBi sample with 1.0% Bi.** The energy values were extracted by using the multi-peak Gaussian fitting of the PL spectra at various temperatures.

To learn more about the PL signals, PL measurements in the temperature range of 8 to 300 K were conducted. Figure 5 shows PL spectra at various temperatures for InPBi with *x*_Bi_ = 1.0%. The PL peak intensity is only enhanced about six times when the temperature decreases from 300 to 8 K. The PL spectra seem to contain multi-peaks, so Gaussian fitting was implemented to extract those multi-peaks and their temperatures dependence was shown in Figure 6. Three overlapped peaks are identified in the PL spectra at *T* < 180 K, whereas at *T* > 180 K the peak at around 0.95 eV disappears and the other two peaks are overlapped. The peak energies labeled peaks 1 and 2 red shifted about 82 and 108 meV, respectively, when the temperature increases from 8 to 300 K, comparable to the red-shifted value of 71 meV for the InP reference sample. However, the peak energies labeled peak 3 are almost constant at around 0.95 eV at various temperatures. To our knowledge, the PL signal of dilute bismides far from the band-to-band transition was scarcely reported in the past. Marko et al. observed the clear and broad PL signal of InGaAsBi sample from 0.46 eV (2.7 μm) to 0.65 eV (1.8 μm) with a much longer wavelength than the band-to-band PL at 0.786 eV (1.6 μm) and attributed to the compositional inhomogeneity [19]. They suggested that the localized narrower-gap regions trapped carriers at low temperatures and produced the long wavelength emission. However, they could only observe the long wavelength PL at *T* < 160 K, and the PL intensity dropped rapidly with temperature, which contrasts to our results. In addition, transmission electron microscope and secondary ion mass spectrometry measurements (not shown here) have revealed quite uniform Bi contents in our InPBi samples. Another possible explanation is that the long wavelength PL is from the recombination related to deep energy levels. The Bi incorporation at low growth temperatures may introduce Bi-related defects such as Bi-antisites [20], which could act as a deep recombination center. Note that the band-to-band PL of InPBi was not observed even at 8 K in our experiments. This suggests a very short carrier lifetime at the bandgap and a long carrier lifetime at the deep levels. Therefore, the origin of the PL signals is still unclear at present, and further investigations are needed to fully account for this phenomenon.

## Conclusions

The structural and optical properties of 430-nm-thick InPBi thin films have been investigated. The Bi compositions determined by RBS measurements were in the range of 0.6% to 2.4%. A good quality has been demonstrated for the samples with the Bi composition lower than 1.4%, whereas the samples with higher Bi contents become partially relaxed. It was found that the incorporation of Bi caused the bandgap reduction of about 56 meV/Bi%. Strong and broad PL signals containing multiple overlapped peaks were observed at room temperature with peak wavelength that varied from 1.4 to 1.9 μm, which is far from the band-to-band transition. The origins of the long wavelength PL signals were discussed, but further investigation is necessary for unambiguous explanation.

## Competing interests

The authors declare that they have no competing interests.

## Authors’ contributions

YG carried out the optical measurements, analyzed the results, and wrote the manuscript. KW grew the samples and performed XRD measurements. HFZ, YYL, CFC, and LYZ helped in the measurements and analysis of results. YGZ supervised the PL experiments and revised the manuscript. QG supervised the growth and joined the discussions. SMW proposed the initial work, supervised the sample design and analysis, and revised the manuscript. All authors read and approved the final manuscript.
